# Post-chikungunya chronic inflammatory rheumatism: results from a retrospective follow-up study of 283 adult and child cases in La Virginia, Risaralda, Colombia

**DOI:** 10.12688/f1000research.8235.2

**Published:** 2016-04-04

**Authors:** Alfonso J. Rodriguez-Morales, Andrés F. Gil-Restrepo, Valeria Ramírez-Jaramillo, Cindy P. Montoya-Arias, Wilmer F. Acevedo-Mendoza, Juan E. Bedoya-Arias, Laura A. Chica-Quintero, David R. Murillo-García, Juan E. García-Robledo, Juan D. Castrillón-Spitia, Jose J. Londoño, Hector D. Bedoya-Rendón, Javier de Jesús Cárdenas-Pérez, Jaime A. Cardona-Ospina, Guillermo J. Lagos-Grisales

**Affiliations:** 1 Public Health and Infection Research Group, Faculty of Health Sciences, Universidad Tecnológica de Pereira, Pereira, Colombia; 2 Organización Latinoamericana para el Fomento de la Investigación en Salud (OLFIS), Bucaramanga, Colombia; 3 Committee on Zoonoses and Haemorrhagic Fevers, Asociación Colombiana de Infectología (ACIN), Bogota, Colombia; 4 Working Group on Zoonoses, International Society for Chemotherapy, Aberdeen, UK; 5 School of Medicine, Faculty of Health Sciences, Universidad ICESI, Cali, Colombia; 6 ESE Hospital San Pedro y San Pablo, La Virginia, Colombia

**Keywords:** Chikungunya, Chronic complications, Epidemiology, Colombia

## Abstract

*Objective: *There are limited studies in Latin America regarding the chronic consequences of the Chikungunya virus (CHIK), such as post-CHIK chronic inflammatory rheumatism (pCHIK-CIR). We assessed the largest cohort so far of pCHIK-CIR in Latin America, at the municipality of La Virginia, Risaralda, a new endemic area of CHIK in Colombia.

*Methods:* We conducted a cohort retrospective study in Colombia of 283 patients diagnosed with CHIK that persisted with pCHIK-CIR after a minimum of 6 weeks and up to a maximum of 26.1 weeks. pCHIK cases were identified according to validated criteria via telephone.

*Results:* Of the total CHIK-infected subjects, 152 (53.7%) reported persistent rheumatological symptoms (pCHIK-CIR). All of these patients reported joint pains (chronic polyarthralgia, pCHIK-CPA), 49.5% morning stiffness, 40.6% joint edema, and 16.6% joint redness. Of all patients, 19.4% required and attended for care prior to the current study assessment (1.4% consulting rheumatologists). Significant differences in the frequency were observed according to age groups and gender. Patients aged >40 years old required more medical attention (39.5%) than those ≤40 years-old (12.1%) (RR=4.748, 95%CI 2.550-8.840).

*Conclusions:* According to our results, at least half of the patients with CHIK developed chronic rheumatologic sequelae, and from those with pCHIK-CPA, nearly half presented clinical symptoms consistent with inflammatory forms of the disease. These results support previous estimates obtained from pooled data of studies in La Reunion (France) and India and are consistent with the results published previously from other Colombian cohorts in Venadillo (Tolima) and Since (Sucre).

## Introduction

Chronic rheumatologic sequelae after Chikungunya virus infection (CHIK) are expected to become an important public health issue in the new endemic areas of Latin America, where the virus has been spreading without proper control ^1^. Previous estimates have shown that nearly 48% of people affected develop post-CHIK chronic inflammatory rheumatism (pCHIK-CIR) ^2^, with the derived burden of disease assessed by disability adjusted life years (DALYs) lost. In Latin American countries these DALYs are now consistently higher than those reported in previous epidemics in India in 2006. DALYs in Colombia are high as 2/3 of those for ischemic heart disease and are related mainly to chronic rheumatologic sequelae ^3– 5^.

However, previous estimates were the result of pooled data from prior studies conducted in India and La Reunion (France) epidemics ^2^. Due to possible virus lineage disparity, different host genetic or immune response, or even environmental variations, those results could not be fully extrapolated to Latin American countries. For these reasons, two retrospective cohorts have been published from Colombia and Latin America, reporting a higher proportion of patients evolving to pCHIK-CIR, particularly chronic polyarthralgia (pCHIK-CPA). Unfortunately, in those studies, the number of patients followed and the assessment of clinical inflammatory characteristics is still limited ^6, 7^.

In this setting, there is still a need to continue assessment and establish the proportion of patients evolving to pCHIK-CIR, both CPA and chronic arthritis (pCHIK-CA), in Latin American countries in order to reveal the real scenario we could face in terms of clinical consequences and chronic disability. Hence, we assessed this issue in the municipality of La Virginia in the department of Risaralda, a new endemic area of CHIK in Colombia, where autochthonous transmission and cases have been detected since January 2015.

## Methods

Ethics approval was obtained from the ESE Hospital IRB.

**Figure 1.**
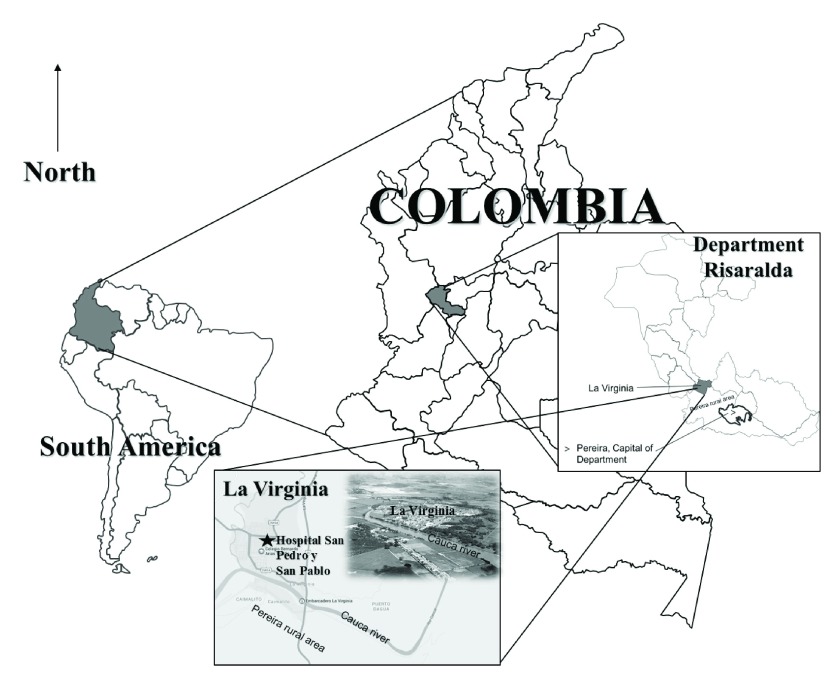
Study area, La Virginia city/municipality, Risaralda department, Colombia, South America.

This retrospective cohort study included patients that suffered CHIK (diagnosed by positive CHIK specific serology, IgM/IgG anti-CHIK, and negative serology for dengue) between February–June 2015 attending in La Virginia, Risaralda (one of the newly endemic departments), Colombia ( Figure 1), with at least 6 weeks between diagnosis and minimal follow-up time for reassessment. The primary outcome was the development of persistent polyarthralgia (pCHIK-CPA) that met the American College of Rheumatology/European League Against Rheumatism 2010 criteria for (seronegative) Rheumatoid Arthritis (RA) ^8^. The patient report of articular inflammatory clinical symptoms was assessed. This included: morning stiffness, joint edema and joint redness. This was assessed via phone calls made by trained authors of this study. A structured questionnaire, previously employed in other studies ^6, 7^ was used (see Dataset 1, which includes all the variables assessed in these patients).

Case definition of acute CHIK infection was made according to the National Institute of Health, Bogotá, Colombia, including serology plus fever (temperature ≥39°C) and polyarthralgia or arthritis. Patient consent was sought at the beginning of the interview.

Clinical assessment included interrogatory and physical examination on initial suspicion of CHIK. Following this, blood tests were performed in order to assess by RT-PCR and serology for IgG and IgM for chikungunya.

Relative frequency of pCHIK-CPA as well other pCHIK chronic rheumatological manifestations (morning stiffness, joint edema and joint redness) were assessed overall, by sex, and by age groups estimating the relative risk (RR) with the corresponding 95% confidence intervals (95%CI). Furthermore, using censoring time (the status at last observation for the time in which was assessed), a pCHIK-CPA curve was drawn using the Kaplan-Meier method to describe the pCHIK-CPA persistence time, expressed in weeks. Also a Cox regression was performed to assess differences according age groups and sex in the outcome by time, estimating the hazard ratio (HR) and its corresponding 95%CI.

All data were recorded in a predesigned format, tabulated and the results analyzed statistically by SPSS® statistical software (version 20).

## Results

Raw data for ‘Post-chikungunya chronic inflammatory rheumatism: results from a retrospective follow-up study of 283 adult and child cases in La Virginia, Risaralda, Colombia’ Click here for additional data file. Copyright: © 2016 Rodriguez-Morales AJ et al. 2016 Data associated with the article are available under the terms of the Creative Commons Zero "No rights reserved" data waiver (CC0 1.0 Public domain dedication).

A total of 283 subjects consented to participate. In total, this group was comprised of 173 (61%) women and 110 (39%) men, with a median age of 29.0 years (IQR 17.0–42.0), 84% of whom were from La Virginia, Risaralda, Colombia. All patients presented with fever and acute polyarthralgia. These cases were diagnosed between February and June 2015, with a median follow-up of 9.7 weeks (2.3 months) and a maximum time of 26.1 weeks (6.1 months).

Out of the total CHIK-infected subjects, 152 (53.7%) reported persistent rheumatological symptoms (pCHIK-CIR) ( Table 1). All of these patients reported joint pains (pCHIK-CPA). Regarding symptoms consistent with pCHIK-CA, 49.5% presented morning stiffness, 40.6% joint edema and 16.6% joint redness. Of those with pCHIK-CPA, 19.4% required and attended for care before the current study assessment (1.4% consulting rheumatologists). Among those who presented pCHIK-CIR, the maximum censored persistence time was 26.1 weeks (6.1 months). From the total, 17 patients where followed ≥20 weeks, and 13 of these patients (76.5%) are still presenting with pCHIK-CPA.

**Table 1.** Prevalence of pCHIK-CPA in a cohort of La Virginia, Risaralda, Colombia; overall, by gender, and by age group.

% pCHIK-CPA	Sex			
Age (years old)	Female (n=173)	Male (n=152)	Total	RR (95%CI)
**<20.000 (n=88)**	29.8	24.4	27.3	1.221 (0.609–2.447)
**20.000–24.999** **(n=32)**	38.9	57.1	46.9	0.681 (0.326–1.420)
**25.000–29.999** **(n=22)**	66.7	53.8	59.1	1.238 (0.625–2.452)
**≥30.000** **(n=141)**	76.8	57.1	70.9	1.343 (1.012–1.784)
**Total**	59.5	44.5	53.7	1.337 (1.049–1.703)

RR=Relative risk; 95%CI=95% confidence interval; pCHIK-CPA=post-Chikungunya chronic polyarthralgia.

The cumulative prevalence of pCHIK-CPA varied significantly (p=0.0326) between those aged >40 years old (69.7%) and those ≤40 years old (47.8%) (RR=1.46, 95%CI 1.04–2.04). As expected, frequency increased with age group ( Table 1). There was also a significant difference (p=0.014) between genders, with pCHIK-CPA being higher in women (59.5%) than in men (44.5%) (RR=1.337, 95%CI 1.049–1.703) ( Table 1), which was also seen among those ≥30 years old ( Table 1).

**Figure 2.**
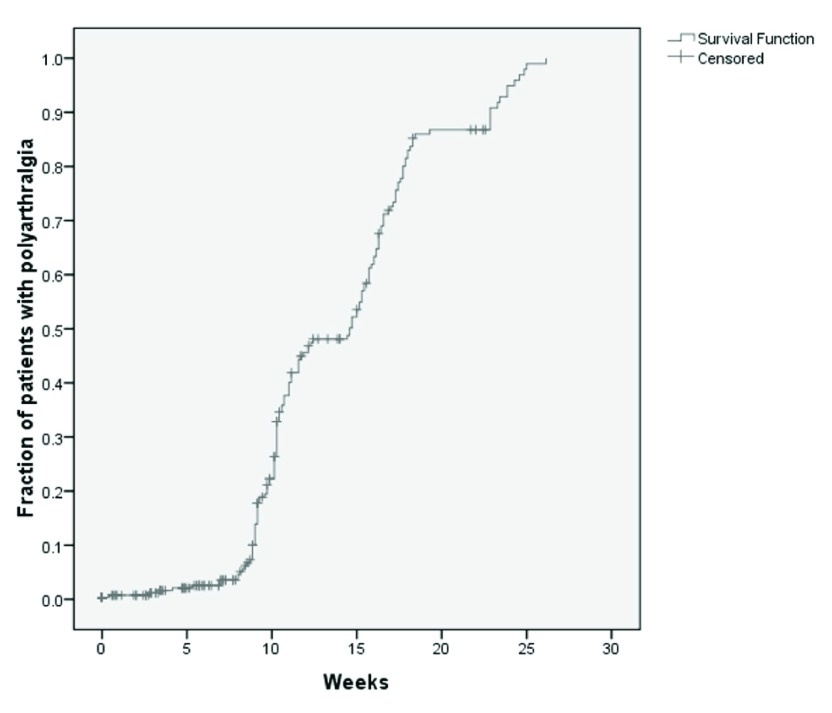
Kaplan-Meier curve of the cumulative prevalence of pCHIK-CPA by follow-up time.

A cumulative prevalence of pCHIK-CPA curve was drawn using the Kaplan-Meier method to describe the pCHIK-CPA persistence time ( Figure 2). After the follow-up, only 46.3% patients remain free of polyarthralgia. The median time for pCHIK-CPA in this cohort was 14.6 weeks (>3 months) (95%CI 12.3–16.8). No significant difference (p>0.05) in the survival function according to age group (HR=1.086, 95%CI 0.774–1.523) or gender (HR=1.086, 95%CI 0.753–1.495) was observed.

Other pCHIK chronic rheumatological symptoms also varied significantly by age. Morning stiffness in those aged >40 years old (60.5%) was significantly higher (p=0.024) than in those ≤40 years old (45.4%) (RR=1.843, 95%CI 1.079–3.148). Joint edema also was significantly higher in those aged >40 years old (53.9%) compared to those ≤40 years old (35.7%) (RR=2.105, 95%CI 1.235–3.588). Patients aged >40 years-old required more medical attention (39.5%) than those ≤40 years old (12.1%) (RR=4.748, 95%CI 2.550–8.840). As expected, there was a trend of frequency increasing by age groups with significant differences between females and males in some of them ( Table 2).

**Table 2.** Prevalence of other pCHIK chronic rheumatological symptoms in a cohort of La Virginia, Risaralda, Colombia; overall, by gender, and by age group.

Rheumatological symptoms	Age group (years old)	Sex					
		Female (n=173)	Male (n=152)	Total	RR	95%CI	
Redness	<20.000 (n=88)	8.5	9.8	9.1	0.872	0.233	3.269
	20.000–24.999 (n=32)	16.7	14.3	15.6	1.167	0.225	6.058
	25.000–29.999 (n=22)	11.1	23.1	18.2	0.481	0.059	3.922
	≥30.000 (n=141)	26.3	9.5	21.3	**2.758**	**1.026**	**7.413**
	Total	19.7	11.8	16.6	1.663	0.919	3.008
Stiffness	<20.000 (n=88)	29.8	22.0	26.1	1.357	0.657	2.802
	20.000–24.999 (n=32)	44.4	35.7	40.6	1.244	0.520	2.977
	25.000–29.999 (n=22)	77.8	53.8	63.6	1.444	0.783	2.665
	≥30.000 (n=141)	70.7	47.6	63.8	**1.485**	**1.055**	**2.089**
	Total	57.2	37.3	49.5	**1.535**	**1.167**	**2.020**
Edema	<20.000 (n=88)	21.3	17.1	19.3	1.246	0.522	2.976
	20.000–24.999 (n=32)	55.6	21.4	40.6	2.593	0.876	7.671
	25.000–29.999 (n=22)	66.7	23.1	40.9	2.889	0.967	8.633
	≥30.000 (n=141)	61.6	35.7	53.9	**1.725**	**1.117**	**2.664**
	Total	50.3	25.5	40.6	**1.976**	**1.389**	**2.810**
Medical	<20.000 (n=88)	8.5	9.8	9.1	0.872	0.233	3.269
Consultations	20.000–24.999 (n=32)	11.1	0.0	6.3	N/A		
	25.000–29.999 (n=22)	22.2	0.0	9.1	N/A		
	≥30.000 (n=141)	39.4	9.5	30.5	**4.136**	**1.578**	**10.842**
	Total	27.2	7.3	19.4	**3.736**	**1.836**	**7.602**

RR=Relative risk; 95%CI=95% confidence interval; pCHIK=post-Chikungunya.

From the total of patients, 38.2% presented polyarthralgia and morning stiffness simultaneously. Polyarthralgia and morning stiffness was this significantly higher in those aged >40 years old (52.6%) was significantly higher (p=0.002) than in those ≤40 years old (32.9%) (RR=1.418, 95%CI 1.098–1.830) as well in females (46.2%) compared with males (25.5%) (RR=1.817, 95%CI 1.270–2.598); 11.3% presented also redness with polyarthralgia and morning stiffness simultaneously, being this significantly higher in females (15.6%) was significantly higher (p=0.004) than in males (4.5%) (RR=3.434, 95%CI 1.363–8.649); 9.9% presented joint edema, redness, polyarthralgia and morning stiffness simultaneously, being this significantly higher (p=0.005) in females (13.9%) was significantly higher than in males (3.6%) (RR=3.815, 95%CI 1.360–10.699).

Finally, in patients <20 years old, >20% of patients in each age group presented with pCHIK-CPA ( Table 3); in general pCHIK-CPA prevalence was higher in female patients, with the exception of the <10 years old age group where 31.3% of males presented with pCHIK-CPA ( Table 3).

**Table 3.** Prevalence of pCHIK-CPA in those <20 years old.

% pCHIK-CPA	Sex			
Age (years-old)	Female (n=47)	Male (n=41)	Total	RR (95%CI)
**<10.000 (n=29)**	15.4	31.3	24.1	0.492 (0.113–2.136)
**10.000–14.999** **(n=28)**	25.0	18.8	21.4	1.333 (0.324–5.486)
**15.000–19.999** **(n=31)**	40.9	22.2	35.5	1.841 (0.491–6.901)
**Total (n=88)**	29.8	24.4	27.3	1.221 (0.609–2.447)

RR=Relative risk; 95%CI=95% confidence interval; pCHIK-CPA=post-Chikungunya chronic polyarthralgia.

## Discussion

According to our results, at least half of the patients with CHIK could develop chronic rheumatologic sequelae, and from those with pCHIK-CPA, nearly half could present clinical symptoms consistent with inflammatory forms of the disease. These results support previous estimates obtained from pooled data of studies in La Reunion (France) and India ^2^ and are consistent with the results published before from other Colombian cohorts in Venadillo (Tolima) and Since (Sucre) ^6, 7^. Indeed, our results suggest that the proportion of patients developing pCHIK-CIR could be even higher in some groups, especially women and older patients. Some patients persisted with rheumatism many months after CHIK infection. Furthermore, the symptoms led nearly 20% of patients to seek care, with some patients requiring attention by a rheumatologist. This highlights the incapacitating character of the symptoms.

This study represents the largest cohort of CHIK patients in Latin America followed to-date for chronic rheumatologic sequelae. Also, this is the first study to assess the development of both inflammatory and non-inflammatory rheumatologic complications post-CHIK infection in this region; previous local studies centered on the presence of CPA without examining clinical inflammatory characteristics such as morning stiffness, joint edema or joint redness ^6, 7^. The epidemiology of the CHIK epidemic in 2015 ^9^ highlights the importance of the assessment of pCHIK-CIR, which should be included in the surveillance of endemic countries such as Colombia.

Although limited due to the possible bias derived from the way in which information was collected (telephone interview), the consistency of the findings related to the proportion of patients that presented CPA and articular inflammatory symptoms with the variables of age and gender, supports the reliability of the obtained data. Smaller studies (<150 patients) recently have also employed telephone interviews to collect data ^10^. One such study found that one third (37%) of study participants reported ongoing complaints related to Chikungunya including joint pain (32%), muscle pain (32%), and joint swelling (26%). A presumptive diagnosis of pCHIK chronic inflammatory arthritis (n = 4) and pCHIK musculoskeletal disorder (n = 3) was established ^10^.

However, the absence of confirmation of chronic articular inflammatory signs by a physician, the lack of information regarding the previous rheumatologic history, number and location of the ongoing articular involvement, received treatment (both prescribed and self-provided), and laboratory and radiographic assessment are important limitations of this work.

While some studies have failed to demonstrate any factors associated with chronic pain ^11^, other works have pointed to risks factors linked to the persistence of chronic rheumatologic manifestations and lack of recovery. In this study older age and female gender where significantly associated with pCHIK-CIR. Nevertheless, in prospective studies, severe initial joint pain, longer acute stage, underlying rheumatologic disease, added comorbidities, overweight and higher IgG anti-CHIK titers, should be also assessed as has been suggested ^12– 16^. Moreover, we also found that patients older than 40 years old and females consulted more frequently and tended to exhibit inflammatory symptoms, which raises concern about the possibility that those patients not only evolve more frequently to pCHIK-CIR but to more severe forms of the disease such as pCHIK-CA. However, also of great concern, is the fact that pCHIK-CIR was seen in a considerable proportion (almost 25%) of young people (<20 years old). Although the current study was not specifically designed to assess pCHIK-CPA in the pediatric population, it shows pCHIK-CPA occurrence in this age group for the first time in Colombia and Latin America. Globally, there is also a lack of cohort studies assessing pCHIK-CIR in children. Prior to this work, the largest study included 69 children <16 years old ^17^; we assessed 88 patients <20 years old (57 <15 years old). This age group also deserves and requires specific studies assessing pCHIK-CIR given all the potential implications, including assessment by pediatric rheumatologists.

Previous cohorts, from France and India, have reported prevalences of pCHIK-CIR ranging from 4.4% to 81.1% with different follow up times (between 2.5 to 72 months) and different assessment procedures, including in-person, via telephone, imaging and laboratory ^11, 14, 17– 21^. Estimations from those studies showed a prevalence of pCHIK-CIR of 41.57% (95%CI 45.08–50.13) in a median time of 20.12 months ^2^. And, in the two previous published cohorts from Colombia the proportion ranged from 44.3 to 89.7% ^6, 7^. Hence, the real risk of developing chronic rheumatologic forms of the disease remains unclear and, despite the fact that bone and articular erosions have been reported even after three years of follow up ^18^, the duration of the articular involvement remains uncertain. The proportion of patients that develop inflammatory forms of the disease needs further assessments. Recent studies have found that pCHIK-CIR can persist even after 6 years of follow-up, as was the case for 59% of patients from 2006–2012 in La Reunion, France ^20^. Nonetheless, our findings are consistent with previous results and certainly raise concern about the future burden of CHIK since assessment using these estimates is already high ^3, 4, 22, 23^.

Besides that, the treatment of inflammatory pCHIK-CIR in Colombia is currently guided by the recommendations to treat rheumatoid arthritis with the consequent economical burden to the health system ^24^. However, there is a lack of high quality evidence to guide treatment and to reassure risk reduction of musculoskeletal sequelae, although non-steroidal anti-inflammatory drugs and disease modifying anti-rheumatic drugs have shown good results in previous works ^25, 26^.

Hence, more studies are needed, particularly prospective studies in order to clarify risk factors, clinical forms, derived disability from rheumatologic symptoms, evolution and treatment. CHIK spread remains without proper control in Latin America, the development of an effective vaccine does not seem like a plausible scenario in the near future and the more expeditious way to mitigate its spread seems to be vector control and educational strategies. If policy makers do not address the problem of disease spread and research needs in this area, cost and burden of chronic sequelae could debilitate already fragile health systems. Recently the National Institute of Health of Colombia (Instituto Nacional de Salud, Bogotá), have estimated that the 817,442 cases (August 2014–August 2015) cost around US$ 25.4–384.2 million (currency change of September 15, 2015) (US$ 31–470 per case, just considering acute phase) ^27^. However, data from our group, recently published ^28^, considering both, acute and chronic phases (estimating the number of patients that will persist with pCHIK-CIR) ^2^, have found that 106,592 cases (August 2014–December 2014) cost around US$ 65.1–164.2 million (US$ 611–1540 per case) ^28^. This represents very high costs for the country, even in the most conservative scenario.

More health authorities frankly need to consider the importance of surveillance for chronic patients in this setting. Governments must face the reality that CHIK is a vector borne disease that is likely here to stay, representing more than 5,000 new cases in 2016. Since September 2015, in addition to CHIK, another arthritogenic arbovirus is cocirculating in the country, Zika, needing studies that assess if chronic complication with it are also possible.

Finally, in Colombia ^29^, as well in other countries in the region, the genotype circulating is the Asian. In the future, if other CHIK genotypes begun to circulate in the same areas, comparison would allow to assess if they impose different clinical impacts, as currently assessing this with past epidemics where other genotypes and lineages were present, as the Indian Ocean one, would also imply potential differences in population immunogenetics and responses probably based in HLA and other ethnic factors. This has been recently showed regard the differences of pCHIK-CIR prevalences between studies in La Reunión island, France and India, being higher in the first, as evidenced in a meta-analysis which is coming out in the next weeks from our group ^30^.

## Data availability

The data referenced by this article are under copyright with the following copyright statement: Copyright: © 2016 Rodriguez-Morales AJ et al.

Data associated with the article are available under the terms of the Creative Commons Zero "No rights reserved" data waiver (CC0 1.0 Public domain dedication). 

*F1000Research*: Dataset 1. Raw data for ‘Post-chikungunya chronic inflammatory rheumatism: results from a retrospective follow-up study of 283 adult and child cases in La Virginia, Risaralda, Colombia’., 10.5256/f1000research.8235.d116559 ^31^

## Consent

Written informed consent for publication of their clinical details was obtained from the patients/parents of the patients. The IRB of the hospital approved this study.
