# Supplementary material for: Post-chikungunya chronic inflammatory rheumatism: results from a retrospective follow-up study of 283 adult and child cases in La Virginia, Risaralda, Colombia
Source: F1000Res. 2016 Apr 4;5:360. Originally published 2016 Mar 16. [Version 2] doi: 10.12688/f1000research.8235.2 (PMC4813633; doi:10.12688/f1000research.8235.2)
Supplement: Peer review history

# Peer review: Referee response for version 1

**DOI:** 10.5256/f1000research.8857.r13074

**Published:** 2016-03-29

**Authors:** Jorge L. Alvarado-Socarras

**Affiliations:** 1 Neonatology Unit, Department of Pediatrics, Cardiovascular Foundation of Colombia, Floridablanca, Colombia

It would be interesting in future studies, evaluate Laboratories data, such as rheumatologic profile and autoimmune diseases.

I have read this submission. I believe that I have an appropriate level of expertise to confirm that it is of an acceptable scientific standard.

# Response

**Published:** 2016-03-29

**Authors:** Alfonso Rodriguez-Morales

**Affiliations:** Universidad Tecnológica de Pereira, Colombia

Thanks for your comments. The plan now is to perform prospective cohort studies, including more detailed laboratory findings, such as those related to rhematological profile and autoimmune diseases.

# Peer review: Referee response for version 1

**DOI:** 10.5256/f1000research.8857.r12907

**Published:** 2016-03-24

**Authors:** Lin H. Chen

**Affiliations:** 1 Mount Auburn Hospital, Cambridge, MA, USA

This is a nicely done survey that assessed the persistence of rheumatologic symptoms in patients diagnosed with chikungunya. 

I presume the virus was the Asian lineage (rather than the Indian Ocean lineage that caused the outbreak in La Reunion), so it's interesting to see the impact of different virus lineages. 

The term "censoring time" may need clarification.

I have read this submission. I believe that I have an appropriate level of expertise to confirm that it is of an acceptable scientific standard.

# Response

**Published:** 2016-03-24

**Authors:** Alfonso Rodriguez-Morales

**Affiliations:** Universidad Tecnológica de Pereira, Colombia

Thank you very much for your comments. Regarding the comment on the Asian genotype, this is in fact the one is circulating in Colombia as in Latin America, has been evidenced in different studies. We agree on the possibility of different potential impacts regard genotype. Nevertheless, other genotypes of CHIK are not circulating yet here. But in the future, if occurs, definitively should be assessed. Then, considering this, we have included a comment on it at the discussion, including a pertinent supporting reference, as well new information from a recently accepted meta-analysis from our group at Arthritis Care and Research about pCHIK-CIR:

*"Finally, in Colombia ^29^, as well in other countries in the region, the genotype circulating is the Asian. In the future, if other CHIK genotypes begun to circulate in the same areas, comparison would allow to assess if they impose different clinical impacts, as currently assessing this with past epidemics where other genotypes and lineages were present, as the Indian Ocean one, would also imply potential differences in population immunogenetics and responses probably based in HLA and other ethnic factors. This has been recently showed regard the differences of pCHIK-CIR prevalences between studies in La Reunión island, France and India, being higher in the first, as evidenced in a meta-analysis which is coming out in the next weeks from our group ^30^.*

*References:*

*29     Mattar S, Miranda J, Pinzon H, et al.: Outbreak of Chikungunya virus in the north Caribbean area of Colombia: clinical presentation and phylogenetic analysis. J Infect Dev Ctries. 2015;9:1126–32. 26517488 10.3855/jidc.6670*

*30     Rodríguez-Morales AJ, Cardona-Ospina JA, Urbano-Garzón SF, et al.: Prevalence of post-Chikungunya Chronic Inflammatory Rheumatism: A Systematic Review and Meta-Analysis. Arthritis Care Res (Hoboken). 2016. (accepted, in press, ACR-15-0598.R2)"*

Regard, the clarification of "censoring time", although this a pretty standard term in the analyses of survival and Kaplan-Meier curves, we have added a detail on this:

*"Furthermore, using censoring time (the status at last observation for the time in which was assessed),..."*

Thanks again for your constructive comments.

# Response

**Published:** 2016-03-29

**Authors:** Alfonso Rodriguez-Morales

**Affiliations:** Universidad Tecnológica de Pereira, Colombia

References 30 is live online now (as accepted manuscript):

Rodríguez-Morales AJ, Cardona-Ospina JA, Urbano-Garzón SF, Hurtado-Zapata JS. **Prevalence of post-Chikungunya Chronic Inflammatory Rheumatism: A Systematic Review and Meta-Analysis**. *Arthritis Care Res (Hoboken)* 2016 Epub Ahead Mar 25; available online at: http://onlinelibrary.wiley.com/doi/10.1002/acr.22900/abstract (Indexed on Medline/Index Medicus)

# Peer review: Referee response for version 1

**DOI:** 10.5256/f1000research.8857.r12911

**Published:** 2016-03-23

**Authors:** Jaime R Torres

**Affiliations:** 1 Infectious Diseases Section, Tropical Medicine Institute of Caracas, Central University of Venezuela, Caracas, Venezuela

The article is well written and both the design and conclusions are sound. 

In the title, the term "chronic inflammatory rheumatism" is not widely used and should be replaced by "chronic rheumatic manifestations"

I have read this submission. I believe that I have an appropriate level of expertise to confirm that it is of an acceptable scientific standard.

# Response

**Published:** 2016-03-24

**Authors:** Alfonso Rodriguez-Morales

**Affiliations:** Universidad Tecnológica de Pereira, Colombia

Thank you very much for your comments. Regard your recommendation to replace in the current title the expression "chronic inflammatory rheumatism", as would be not widely used and should be replaced by "chronic rheumatic manifestations"; although we truly appreciate your recommendation, we can not proceed according it, because the expression "chronic inflammatory rheumatism" has been used as part of articles title in 105 records in Medline up to March 23, 2016, as can be seen at: https://www.ncbi.nlm.nih.gov/pubmed/?term=chronic+inflammatory+rheumatism+[ti]; conversely, "chronic rheumatic manifestations", has been used just in four: https://www.ncbi.nlm.nih.gov/pubmed/?term=chronic+inflammatory+manifestations+[ti]. Then, is clearly more used "chronic inflammatory rheumatism" than "chronic rheumatic manifestations".
